# Implant-Retained Obturator for an Edentulous Patient with a Hemimaxillectomy Defect Complicated with Microstomia

**DOI:** 10.1155/2016/4618510

**Published:** 2016-10-23

**Authors:** Pravinkumar G. Patil, Smita Nimbalkar-Patil

**Affiliations:** ^1^Division of Clinical Dentistry, School of Dentistry, International Medical University, Kuala Lumpur, Malaysia; ^2^Department of Orthodontics, Faculty of Dentistry, MAHSA University, Kuala Lumpur, Malaysia

## Abstract

*Patient*. A 68-year-old man was operated on for squamous cell carcinoma (T3N3M0) of the maxilla creating the hemimaxillary surgical defect on right side. The remaining arch was completely edentulous. There was remarkable limitation in the oral opening with reduced perimeter of the oral cavity due to radiation and surgical scar contracture. This article describes prosthetic rehabilitation by modifying the design of the obturator and achieving the retention with dental implant.* Discussion*. Severe limitation in the oral opening may occur in clinical situations following the postsurgical management of oral and maxillofacial defects. The prosthetic rehabilitation of the surgical defect in such patients becomes a challenging task due to limited access to the oral cavity. This challenge becomes even more difficult if the patient is edentulous and there are no teeth to gain the retention, stability, and support.* Conclusion*. In severe microstomia prosthesis insertion and removal can be achieved with modification of the maximum width of the prosthesis. Dental implant retention is useful treatment option in edentulous patients with maxillary surgical defect provided that sufficient bone volume and accessibility are there for implant placement.

## 1. Introduction

Microstomia is defined as an abnormally small oral orifice [1]. Head and neck cancer are commonly treated with surgical intervention and radiotherapy [2, 3]. Limited oral opening can be caused by head and neck radiation [3–6], reflex spasm [4], surgically treated head and neck tumors [7], microinvasion of the muscles of mastication [4, 8], connective tissue disease [9], fibrosis of masticatory muscles [10], facial burns [11], and reconstructive lip surgeries [12]. Clinical management of the problems associated with providing dental prostheses for patients with trismus is not well reported [13], although the following management techniques have been described: surgery [4, 14], the use of dynamic opening devices [15], and modification of denture designs [13, 16]. The postsurgical microstomia usually leads to very stiff oral aperture and the stretching of the lips becomes a difficult task during various treatment stages of prosthesis fabrication. To make a primary impression the height of the tray should be smaller than the interarch space and the lips can be stretched to a width that is equal to or greater than the width of an impression tray. In situations where scar tissue formation has decreased the flexibility of the lips significantly, insertion and removal of stock impression trays become difficult. Maxillofacial prosthesis in completely edentulous patient is a challenging task. This article describes the prosthetic management of a patient with an edentulous maxilla and midfacial defect complicated by severe postsurgical microstomia.

## 2. Outline of the Case

### 2.1. Case History

**Figure 1:**
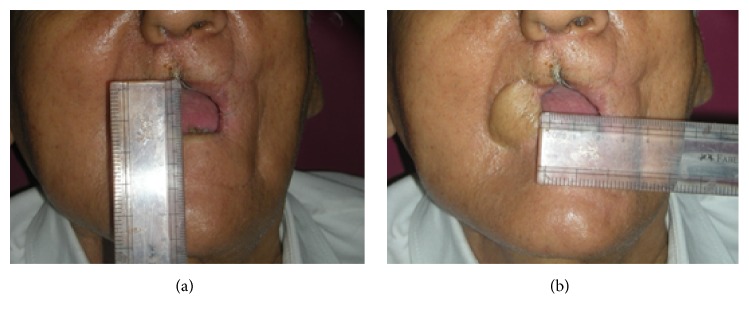
Extraoral view with maximum oral opening with (a) limited vertical length of oral aperture and (b) limited horizontal length of oral aperture.

**Figure 2:**
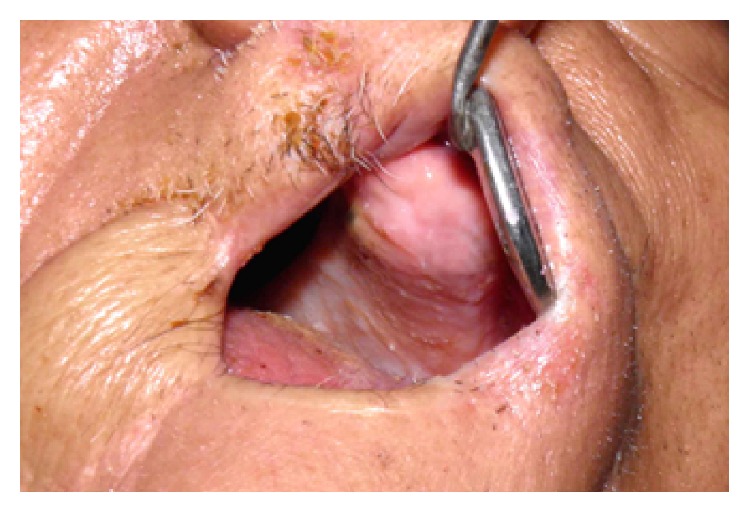
Intraoral view with maximum oral opening. Note the reduced aperture in relation to mouth mirror head.

**Figure 3:**
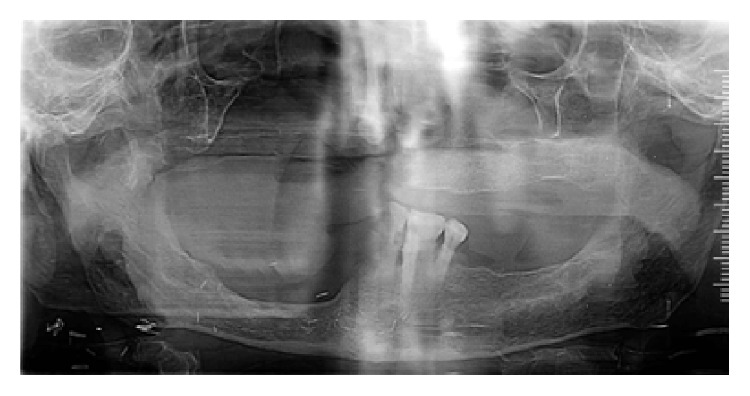
Postsurgical orthopantomograph revealing right side hemimaxillectomy.

A 68-year-old man was referred postsurgically from the ENT Surgery Department to the Department of Prosthodontics for prosthodontic assessment for hemimaxillectomy defect. The patient was frustrated using a gauze-pack since one month and was keen to use any kind of prosthesis that can facilitate his oral feeding and not really concerned with the esthetics. A detailed medical and dental history revealed that he was operated on for the squamous cell carcinoma (T3N3M0) of the right maxilla 1 month ago. The right hemimaxillectomy was performed together with removal of his remaining anterior teeth including central incisor, lateral incisor, and canine on right side and left central incisor. The patient received a preoperative course of total dose of 7200 cGy external beam radiation within a period of 6 weeks in fractions (a fraction of 200 cGy/day for 5 days in a week). The patient was restricted to a liquid-diet through a nasogastric tube for initial 2 weeks after surgery followed by oral liquid feeding for next 2 weeks. He was instructed to use the gauze-pack covered with the polymer sheet (to be replaced twice a day) to block the defect area while taking liquid and semisolid food for next 1 month. Clinical examination revealed a partially edentulous maxilla and mandible. There was remarkable limitation in the oral opening with reduced perimeter of the oral cavity (Figures 1(a) and 1(b)). Intraoral examination revealed healthy maxillary edentulous ridge on left side (Figure 2). Reduced oral aperture size made this case difficult to restore with a conventional obturator prosthesis. Clinical examination revealed that remaining left side of the maxillary arch was completely edentulous and the mandibular arch remained only with left lateral incisor, canine, and first premolar. Postoperative OPG revealed removal of all maxillary teeth along with the right half of the maxilla (Figure 3).

### 2.2. Fabrication of Definitive Obturator

**Figure 4:**
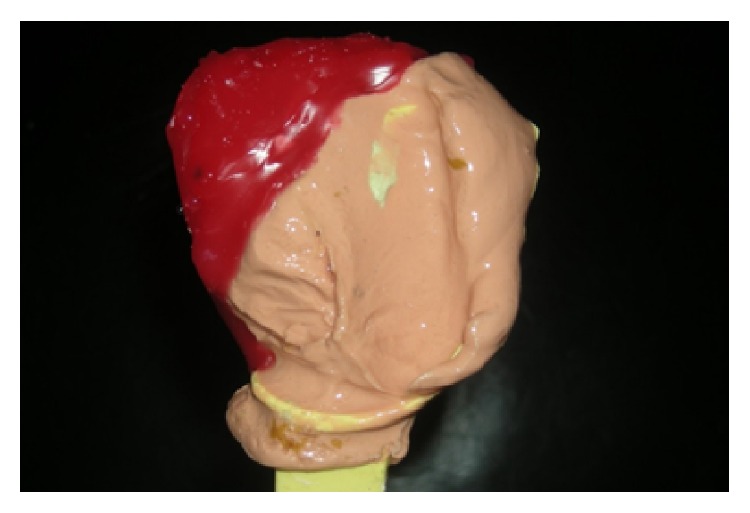
Primary impression recorded with irreversible hydrocolloid using modified perforated plastic-stock-tray. Remaining portion of defect area was built up with modelling wax.

Fabrication of definitive obturator prosthesis was planned. There were three challenges during the obturator fabrication: (1) Remarkable restricted mouth opening and microstomia, which makes standard stock impression trays go inside the mouth; (2) edentulous remaining maxillary arch, which makes the retention of the obturator prosthesis difficult; and (3) difficulty in using mandibular partial denture due to lack of attached mucosa on right side of the mandibular arch. A preoperative diagnostic cast was not available. Due to small aperture size, use of standard sized stock trays was impossible. The plastic perforated stock tray was modified according to the need by trimming the borders and palatal portion of the tray to fit it inside the mouth and the impression was made with irreversible hydrocolloid with the thick mix consistency. The impression was made with the detail records of remaining part of the hard palate and the defect marginal areas along the midpalatine raphe. Remaining portion of the defect area was built up with modelling wax arbitrarily to achieve the natural contour of the palate (Figure 4). The cast was poured in the type III gypsum material (Kalstone; Kalabhai Karson, Mumbai, India). The palatal-obturator-plate was fabricated covering unresected portion and contoured resected portion of the cast in heat polymerized acrylic resin (Heat Cure Acrylic; Dental Products of India, Mumbai, India).

### 2.3. Prosthesis Modification for Facilitation of Insertion/Removal and Retention

**Figure 5:**
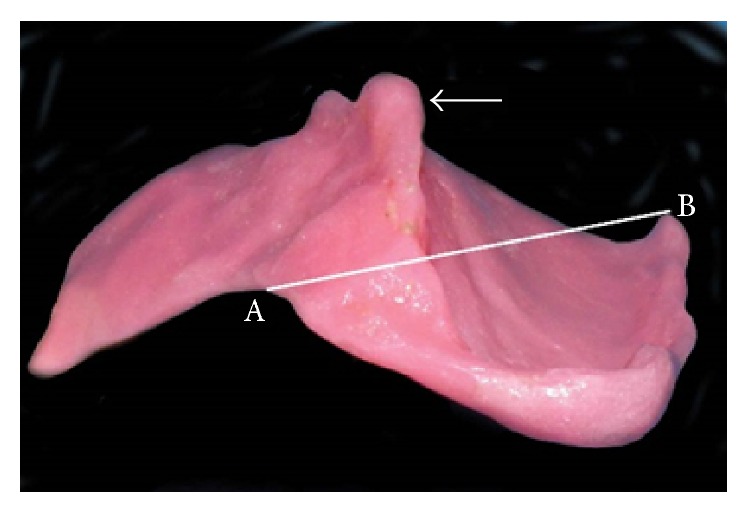
Palatal obturator. Note that arrow indicates extension of the resin in undercut areas seen along the defect margins. Line joining A and B indicates maximum stretchable distance of mouth.

The shape of the palatal obturator was modified to facilitate the easy placement and removal of the prosthesis. The base was made slightly concave along the borders in the region of right canine. This was the area around which the prosthesis was planned to turn inside the mouth during insertion and removal (Figure 3). The point A (see Figure 5) indicates concave area of prosthesis at corner of the mouth allowing insertion of the prosthesis in curvilinear path with centre at point A. The point B (Figure 5) indicates the distobuccal end of prosthesis. The concavity provided at this specific area facilitated the reduction of the maximum horizontal length of the prosthesis which was almost similar to that of maximum stretchable distance of the oral aperture during insertion and removal of prosthesis horizontally. The obturator was placed in the mouth as described earlier and tried to evaluate the retention, stability, and the amount of defect closure area. The obturator was selectively trimmed along the edges to sufficiently close the defect margins without displacement during oral functional movements. As the remaining maxillary arch was edentulous, there was hardly anything to depend upon for retention of the prosthesis except the defect margins along the midpalatal area. This area was relined with the permanent resilient liner (Tokuyama Soft-Plus II; Tokuyama Dental Corporation, Tokyo, Japan) to achieve better adaptation and undercut engagement with the available undercut space along the defect margin. The patient was then evaluated for the need of additional retention during speech and deglutition. The undercut engagement was not sufficient to retain the prosthesis during function as the prosthesis tends to fall especially from anterior aspect. The small test was carried out to identify the need of extra retention. The anterior portion of the prosthesis was stabilized with the one finger and allowed patient to speak and swallow. This time the prosthesis remained in place. So the treatment plan was modified to insert an implant for achieving additional retention with the help of overdenture attachment.

### 2.4. Implant Placement

**Figure 6:**
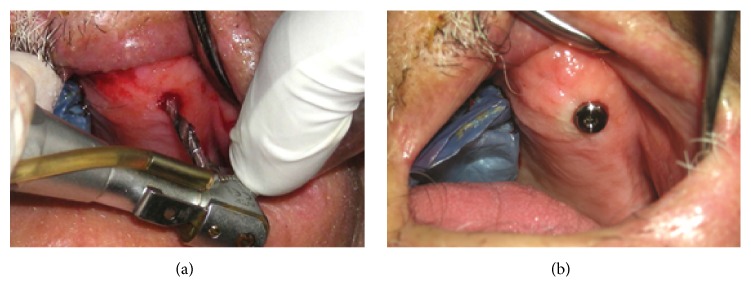
(a) Flapless implant placement. Note limited access of implant surgical hand-piece. (b) Healing abutment in place.

**Figure 7:**
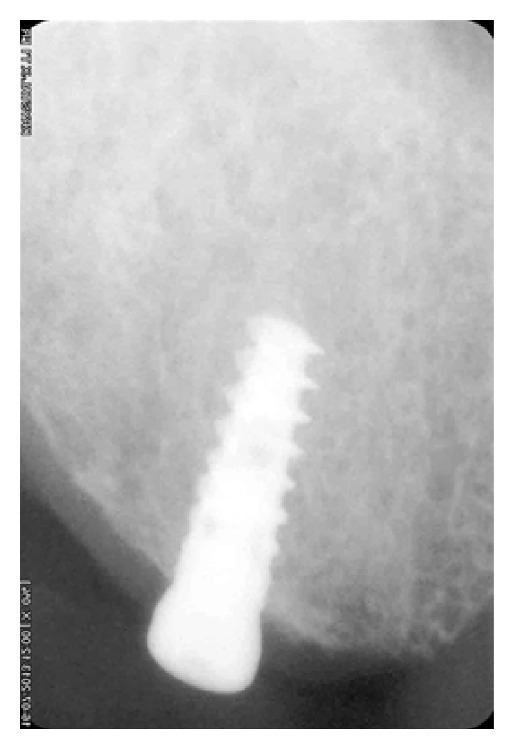
Intraoral periapical radiograph of implant after 4 months of osseointegration.

**Figure 8:**
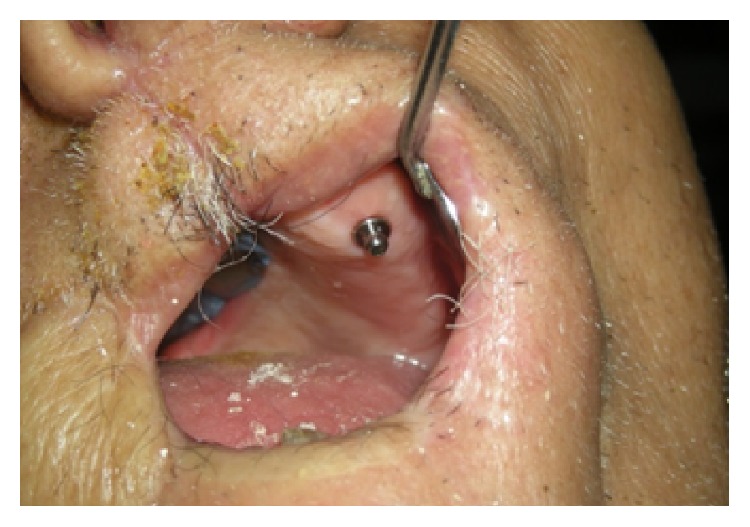
Overdenture attachment in place.

**Figure 9:**
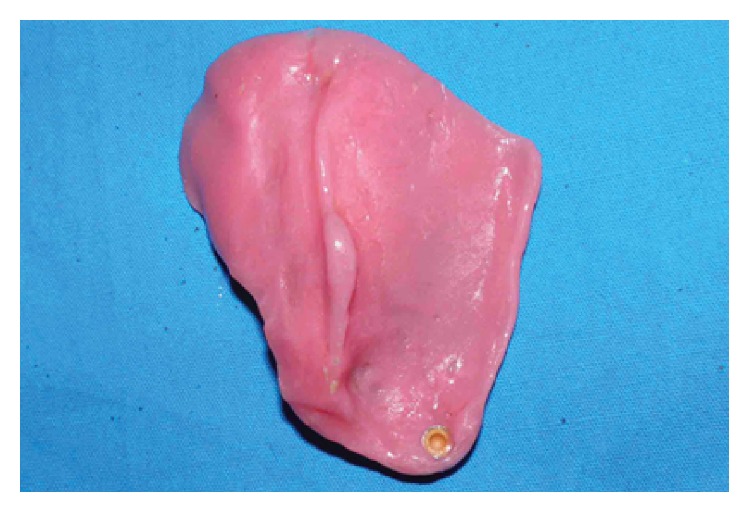
Retentive component incorporated in obturator plate.

The remaining right edentulous maxillary arch was evaluated for the suitability of implant placement. Patient agreed to undergo additional minor surgical procedure of implant placement for the sake of improved retention and stability of the obturator and was convinced for improved eating and swallowing. The site of the implant placement was selected in the anterior most possible area of the right maxilla for implant osteotomy for the implant surgical hand-piece accessibility. The gutta-percha point of 3 mm length was inserted into the same obturator for diagnostic purpose and the periapical radiograph was taken. Bone sounding procedure was carried out in the same region to ensure adequate bone volume. An endosseous self-threading implant of diameter 4.2 mm and length 13 mm (Adin Touareg S; Adin Implant Corporation, Israel) was selected with the help of radiographic evaluation and bone sounding procedure. The flapless surgical procedure was performed by punching out the 3 mm diameter circular attached mucosa to place the implant (Figure 6(a)). The healing abutment was placed to avoid second stage surgery (Figure 6(b)). Four months after surgery, an intraoral periapical radiograph was taken to ensure the osseointegration of the implant (Figure 7). Clinical examination revealed stable implant with healthy peri-implant tissues. The healing abutment was replaced with the overdenture-abutment (ball and socket) (Figure 8). The obturator was then modified from the intaglio surface to accommodate the matrix-components of the attachment relined/fixed with the autopolymerizing acrylic resin (GC Cooliner; GC Corporation, Tokyo, Japan) (Figure 9). The softest available retentive nylon cap along with the metal housing was attached with the direct pick-up technique (Adin overdenture nylon cap with pink colour code). The patient was trained to insert and remove the obturator prosthesis. The possible nasal regurgitation of liquid was evaluated by asking the patient to drink the water. The water regurgitation was found to be reduced and patient could drink with ease compared to previous conditions. The possible leakage areas were identified by evaluating the overextensions and underextensions of the prosthesis margins again and modified accordingly to achieve maximum possible seal during oral functions.

### 2.5. Obturator Delivery and Recall

**Figure 10:**
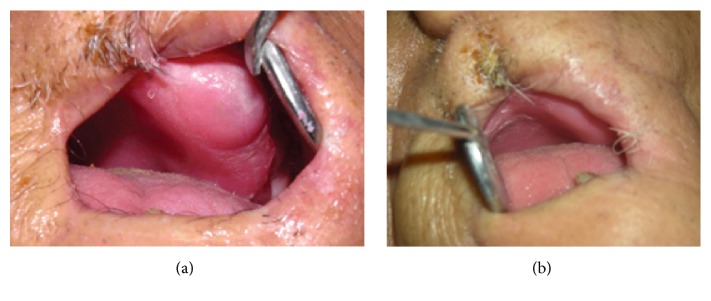
(a) Left lateral view showing implant-retained obturator in place; (b) midpalatal view indicating closed defect.

The patient was not keen to add anterior teeth to the prosthesis as he was more concerned about the functions rather than the esthetics. Hence the teeth were not added to the prosthesis. Patient learned to masticate by contacting his mandibular remaining natural teeth with the anterior portion of the obturator and could manage to handle the semisolid and soft-solid food. The patient was kept under observation for the implant success by taking the periapical radiograph after initial 6 months and then planned to take the periapical radiographs after every 12 months. The soft nylon cap was replaced regularly after every 6 months. The obturator was relined after every 12 months. Last recall visit was after 18 months of the treatment (Figures 10(a) and 10(b)). The marginal bone loss of 1.5 mm was observed around the implant after 18 months of use. The patient was pleased with the improved functions and eating comfort.

## 3. Discussion

Management of postsurgical and/or postradiation patients of oral cancer therapy is a clinically challenging situation. The smaller the defect area is, with healthy teeth present in nonresected portion of the arch, the easier the condition to achieve retention, stability, and support for the prosthesis is. The larger the area is, the harder the prosthodontic rehabilitation is. This problem worsens when the remaining arch is having lesser number of teeth, periodontally compromised teeth or completely edentulous ridge. The implants can be used to gain the retention, stability, and support to the prosthesis in such situation. But the prosthesis movement during functions like speech, mastication, and swallowing makes long-term implant success rate questionable. The careful evaluation of such movement must be done before planning the implant treatment for maxillofacial prostheses. In this patient, fortunately, the prosthesis was seated and adapted well on the remaining edentulous arch with little movement of the obturator during functions. The simple obturator prosthesis (without addition of teeth) was used to obdurate the defect. Addition of teeth would have increased the weight of the prosthesis and the chances of lateral forces to dislodge the prosthesis. The patient's desire to have more importance on oral intake of food than the aesthetic appearance made this prosthesis simpler than the prosthesis with teeth. There was no option other than the implant attachment to achieve retention of the prosthesis due to completely edentulous remaining maxillary arch and inadequate tissue undercut on defect side. However implant placement in the irradiated bone was one of the concerns. Nooh [17] performed a systematic review of the literature with 38 articles published between 1990 and 2012. He concluded that overall implant survival rates with radiation therapy done before and after implantation were 88.9% and 92.2%, respectively. In this patient the implant was survived till 18 months of the last recall and the marginal bone loss was observed to be 1.5 mm, which is considered to be the success of the implant as well.

There are various types of implant overdenture retentive components available. Any overdenture attachment type (including ball/socket, bar/clip, magnets, and locators) can be used as per the clinical situations and treatment needs. We had used the ball and socket with one single implant to allow 360 degrees of freedom to allow the prosthesis to have micromovements in all spatial directions with the retentive component in place. This would probably reduce the unwanted stress on the implant. The single implant was sufficient in this situation to retain the toothless lightweight obturator. Mastication is not possible with this obturator; however patient can eat semisolid and liquid food. The principal purpose of this obturator was to allow liquid/semisolid food intake orally without masticating, as there are no teeth for mastication purpose in lower arch too.

## 4. Conclusion

In severe microstomia prosthesis insertion and removal can be achieved with modification of the maximum width of the prosthesis. Dental implant retention is useful treatment option in edentulous patients with maxillary surgical defect provided that sufficient bone volume and accessibility are there for implant placement.
